# PANoptosis: a potential new target for programmed cell death in breast cancer treatment and prognosis

**DOI:** 10.1007/s10495-023-01904-7

**Published:** 2023-11-24

**Authors:** Xinxin Liu, Meiqi Miao, Jijing Sun, Jianli Wu, Xunyun Qin

**Affiliations:** 1 School of Basic Medical Sciences, Heilongjiang University of Traditional Chinese Medicine, Harbin, 150040 China; 2 Heilongjiang University of Traditional Chinese Medicine, Harbin, 150040 China; 3 Department of Cardiovascular Medicine, The First Affiliated Hospital of Heilongjiang University of Traditional Chinese Medicine, Harbin, 150040 China; 4 Department of Oncology, Beijing Yao Medicine Hospital, Beijing, 100071 China

**Keywords:** PANoptosis, Pyroptosis, Apoptosis, Necroptosis, Breast cancer (BC), Programmed cell death (PCD)

## Abstract

Breast cancer is a prevalent and severe form of cancer that affects women all over the world. The incidence and mortality of breast cancer continue to rise due to factors such as population growth and the aging of the population. There is a growing area of research focused on a cell death mechanism known as PANoptosis. This mechanism is primarily regulated by the PANoptosome complex and displays important characteristics of cell death, including pyroptosis, apoptosis, and/or necroptosis, without being strictly defined by the cell death pathway. PANoptosis acts as a defensive response to external stimuli and pathogens, contributing to the maintenance of cellular homeostasis and overall stability. Increasing evidence suggests that programmed cell death (PCD) plays an important role in the development of breast cancer, and PANoptosis, as a novel form of PCD, may be a crucial factor in the development of breast cancer, potentially leading to the identification of new therapeutic strategies. Therefore, the concept of PANoptosis not only deepens our understanding of PCD, but also opens up new avenues for treating malignant diseases, including breast cancer. This review aims to provide an overview of the definition of PANoptosis, systematically explore the interplay between PANoptosis and various forms of PCD, and discuss its implications for breast cancer. Additionally, it delves into the current progress and future directions of PANoptosis research in the context of breast cancer, establishing a theoretical foundation for the development of molecular targets within critical signaling pathways related to PANoptosis, as well as multi-target combination therapy approaches, with the goal of inducing PANoptosis as part of breast cancer treatment.

## Introduction

**Fig. 1:**
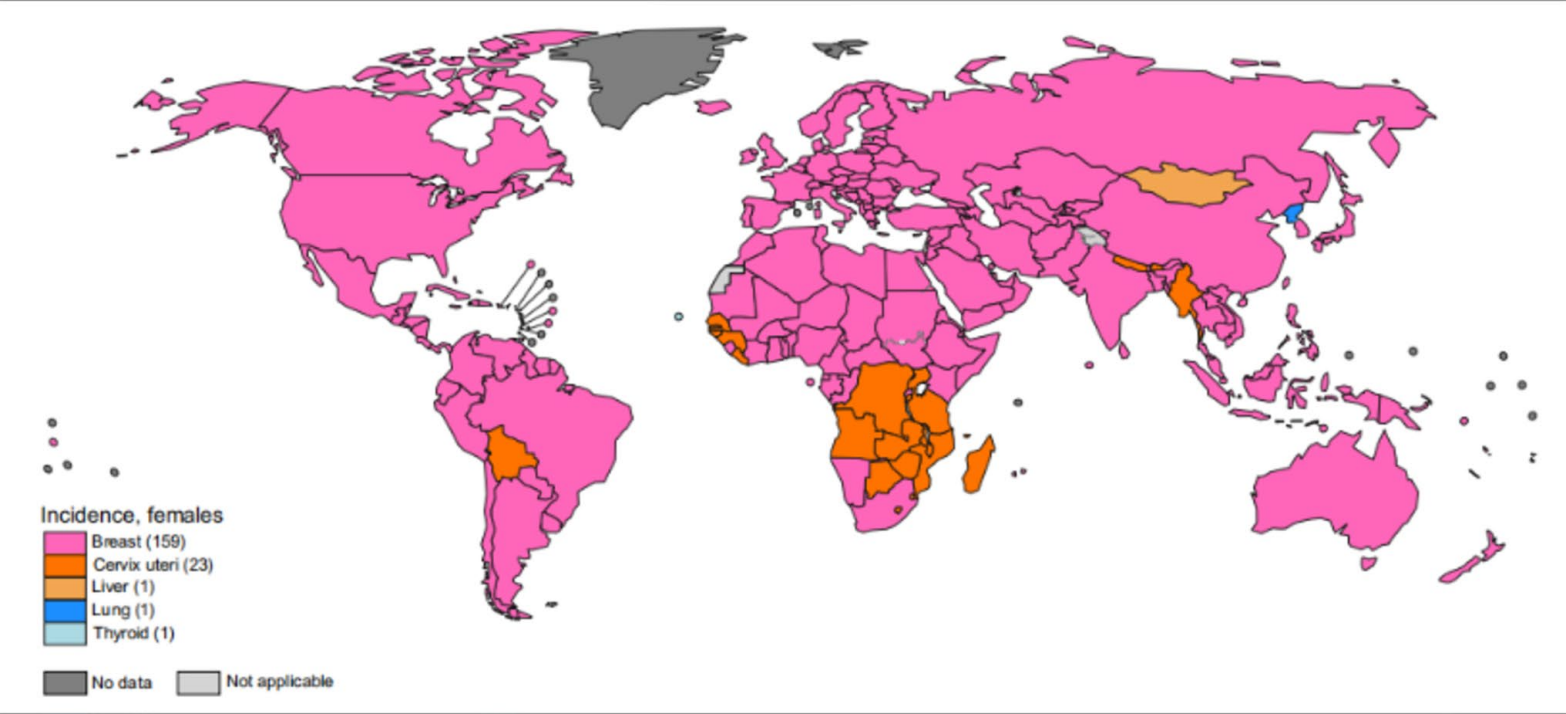
Primary incidence of cancer by country in women for the year 2020 [1]

Breast cancer is a highly prevalent cancer that primarily affects women and is a leading cause of both new cases and deaths among female malignancies worldwide (Fig. 1). According to the statistics in 2020, the global incidence of new breast cancer cases in women was approximately 2.2614 million, accounting for 25.84% of all new cases of female malignancies. The number of deaths was approximately 685,000, accounting for 15.56% of female malignancy-related deaths [1]. The incidence and mortality rates of breast cancer vary significantly among women based on factors such as age and race. In the United States, for instance, it was estimated that in 2022, there would be 339,250 new cases of breast cancer, with 43,250 deaths. A substantial portion of these cases (83%) and deaths (91%) occurred in women aged 50 and above, with half of the deaths occurring in women aged 70 and older. The median age of breast cancer-related deaths was 69 years old, with variations among different ethnic groups, for instance, 70 for White women, 62 for Hispanic women, and 63 for Asian-Pacific Islander and Black women. Additionally, despite lower breast cancer incidence rates among Black women compared to White women, the mortality rate for breast cancer is 40% higher among Black women [2]. By 2023, the incidence of breast cancer in American women is expected to rise to 31%, resulting in an estimated 43,170 deaths, accounting for 7.08% of cancer-related deaths [3]. As the global population continues to expand and age, projections suggest that by 2040, there will be an excess of 3 million fresh breast cancer diagnoses and a tragic toll of 1 million breast cancer-related deaths [4]. Despite the concerning increase in breast cancer incidence at a rate of 0.5% per year from 2010 to 2019, there is a silver lining in the declining mortality rate, which has been decreasing at a rate of 1.3% per year from 2011 to 2020. These statistics indicate significant progress in both the prevention and treatment of breast cancer.

The management of BC is contingent upon distinct molecular subcategories, while the precise origins and mechanisms underlying the ailment remain shrouded in ambiguity. Breast cancer, as a metastatic condition, possesses a propensity for disseminating to distant organs, including brain, lung, liver, and bone, thereby compounding the intricacies of treatment. A spectrum of therapeutic avenues is available for breast cancer, encompassing localized/regional interventions such as surgical procedures and radiation therapy, in conjunction with systemic approaches. Among the systemic approaches are hormone therapy for hormone-positive instances, chemotherapy, anti-human epidermal growth factor receptor 2 (HER2) therapy tailored to HER2-positive cases, and the integration of immunotherapy. Notwithstanding these endeavors, the comprehensive outcomes of treatment still fall short of being deemed satisfactory [5]. Despite the swift strides witnessed in prompt detection and pharmaceutical interventions of late, breast cancer continues to retain its status as the foremost contributor to women's cancer-linked fatalities on a global scale. As a result, there is a significant imperative to investigate new approaches in treatment, predictive factors, and prognostic markers, all aimed at improving patient outcomes and increasing survival rates.

Programmed cell death (PCD) is currently recognized as a strict form of regulatory cell death (RCD) occurring under physiological conditions [6]. This form of RCD is orchestrated by a series of evolutionarily conserved pathways, which hold significant sway over developmental processes and immune reactions [7]. Fundamentally, the instigation and orchestration of RCD predominantly revolve around the creation of complexes that amplify signaling cues.

In the past, apoptosis, necroptosis, and pyroptosis were commonly viewed as distinct forms of PCD. However, recent in-depth investigations into cell death have unveiled substantial mechanistic convergence and intricate interplays among these three pathways [8]. Building upon these discoveries, the concept of “PANoptosis (PAN-optosis)” has been proposed, which represents an inflammatory form of PCD orchestrated by the PANoptosome, a complex governing the execution of death. PANoptosis demonstrates essential attributes of apoptosis, necroptosis, and pyroptosis, yet it defies being pigeonholed into any single mode of cell demise.

Over the years, researchers have uncovered a strong correlation between PCD and the onset as well as the prognosis of breast cancer. As breast cancer advances, instances of cell death frequently manifest within the central regions of solid tumors, attributed to metabolic strain like hypoxia and glucose deficiency [9]. The induction of PCD within these tumors stands as a pivotal strategy in the realm of cancer treatment. Consequently, with the ongoing revelation and characterization of various PCD forms, our comprehension of the role of cell death in tumor contexts is in a constant state of evolution. This review primarily delves into the impact of pan-apoptosis in the emergence and progression of breast cancer, with the intention of offering novel insights to guide future investigations into tumor pathogenesis and the exploration of innovative therapeutic interventions.

## PANoptosis

**Fig. 2:**
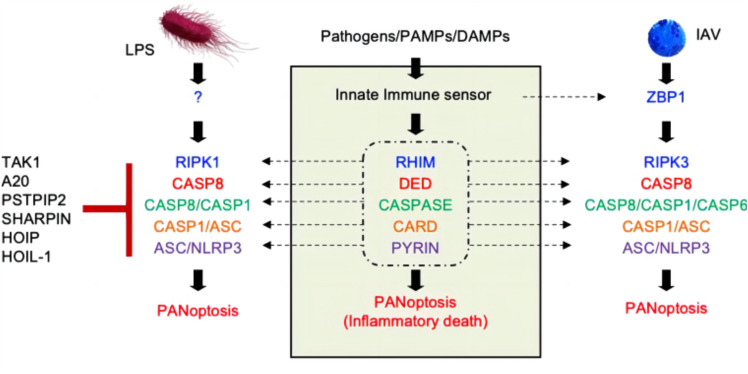
PANoptosis constitutive process [11]. Different stimuli can induce varying compositions of the PANoptosome, but a common feature is that each PANoptosome will have components with critical structural domains, including DEATH domains, DEDs, CARDs, PYRIN domains, and RHIMs, which facilitate the assembly of the PANoptosome

PANoptosis is an inflammatory PCD governed by the PANoptosome complex, encompassing various aspects of pyroptosis (“P”), apoptosis (“A”) and/or necroptosis (“N”). The concept of PANoptosis was first introduced in 2019 by Malireddi's research team. Their proposal underscored the substantial co-regulation and interplay among critical facets of RCD, including pyroptosis, apoptosis and regulatory necrosis (e.g., necroptosis). Additionally, this proposition highlighted the distinctive regulatory pathways inherent to each form of PCD, positing that these shared mechanisms collectively constitute the phenomenon termed PANoptosis [10] (Fig. 2).

### PANoptosome

**Fig. 3:**
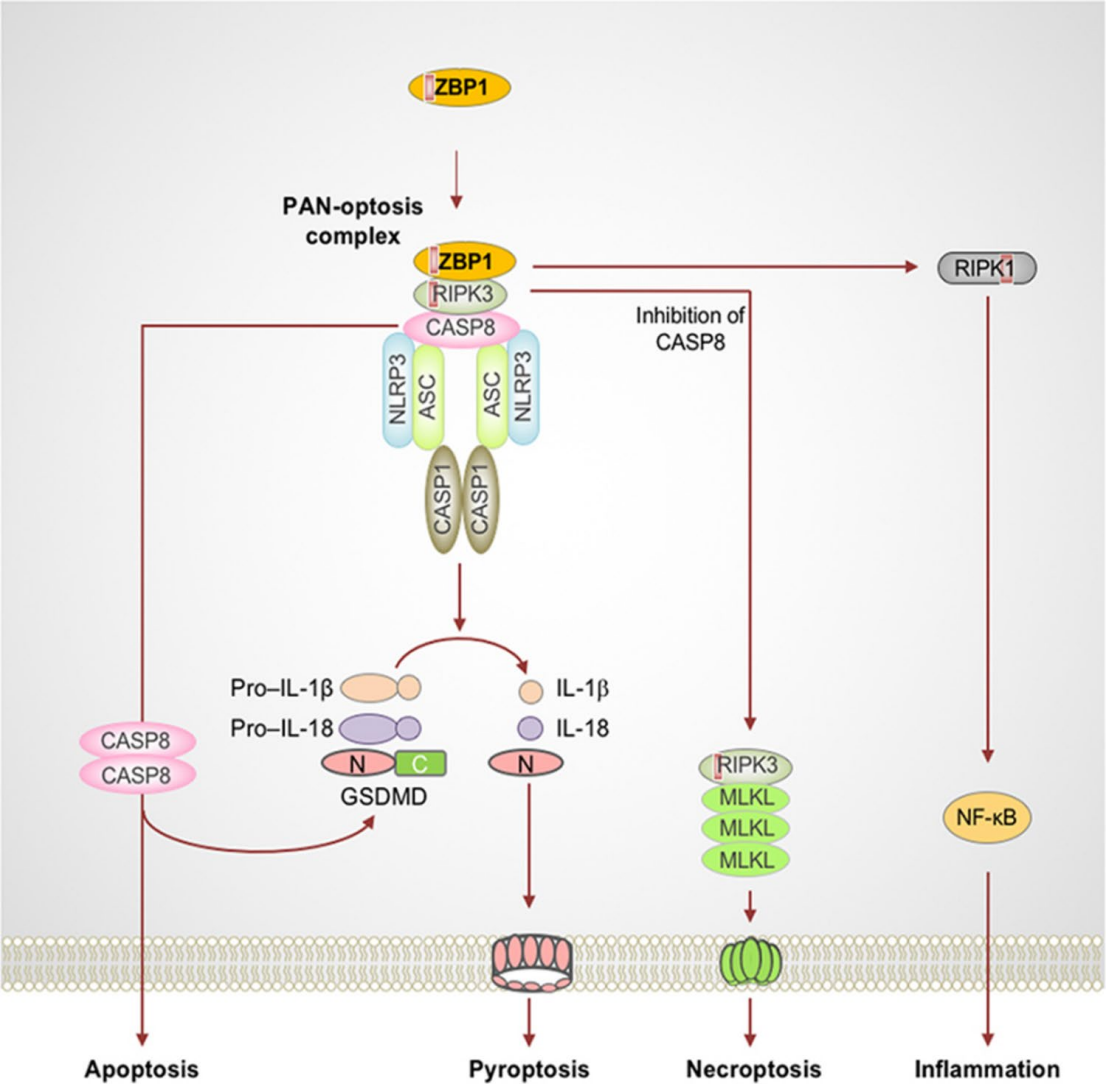
PANoptosis processes involved in ZBP1 and its complexes [9]. ZBP1 activation interacts with the RIPK3 receptor and recruits CASP8 to form a cell death signaling scaffold. This ZBP1-RIPK3-CASP8 complex is involved in NLRP3 inflammasome-dependent thermoapoptosis, CASP8-mediated apoptosis, and RIPK3-MLKL-driven necroptosis. ZBP1 also induces RIPK1-driven NF-κB activation and an inflammatory response during influenza infection. Red boxes within proteins represent RHIM (Color figure online)

The PANoptosome is a multiprotein complex that holds sway over the regulation of apoptotic cell death. However, the exact constituents of the PANoptosome remain incompletely elucidated. Currently available data strongly suggests the pivotal role of Z-DNA Binding Protein 1 (ZBP1) in regulating the “P” (pyroptosis), “A” (apoptosis), and “N” (necroptosis) pathways. ZBP1 serves as a discerning sensor for influenza A virus (IAV) infection, orchestrating its activation that subsequently triggers interactions with Receptor Interacting Serine/Threonine Kinase 1/3 (RIPK1/3). This interaction governs cell death processes, alongside the involvement of caspase-6 (CASP6) and caspase-8 (CASP8), pivotal agents in apoptosis. Collectively, these components amalgamate to shape the ZBP1-PANoptosome. An additional facet of the ZBP1-dependent PANoptosome contributes to the initiation of the NLR Family Pyrin Domain Containing 3 (NLRP3) inflammasome, culminating in gasdermin D (GSDMD)-dependent pyroptosis. When pyroptosis is blocked, activated CASP8 can trigger apoptosis, while inactivation of CASP8 results in mixed lineage kinase domain-like (MLKL)-mediated necroptosis. At present, this compilation of evidence serves as the most comprehensive biochemical substantiation for PANoptosis [10, 11] (Fig. 3). Additionally, a multi-protein complex christened the absent in melanoma 2 (AIM2) PANoptosome, consisting of AIM2, Pyrin, and ZBP1, has been unveiled by Lee et al. [12]. This complex incites PANoptosis during infections spurred by HSV1 and *F. novicida*. In addition, pertubing the activity of TGF-β-activated kinase 1 (TAK1) through gene knockout also leads to pyroptosis, apoptosis, and necroptosis. Notably, TAK1 is a fundamental constituent of the PANoptosome, the complex involved in PANoptosis [13].

Hence, the sequence of events underlying PANoptosis can be succinctly outlined as follows: (1) Initiation: cellular stress or microbial infection, such as IAV, triggers the formation of the PANoptosome through specific triggering factors; (2) Activation of sensors: distinctive sensors (such as ZBP1, AIM2) are prompted into activity by these triggering factors; (3) PANoptosome assembly: the activated sensors trigger the assembly of the PANoptosome, complex encompassing essential molecules crucial for activating downstream effectors responsible for PCD, including MLKL, caspase-3/7 (CASP3/7), and gasdermins [14].

### Activation of cell death

These apoptotic complexes play a pivotal role in catalyzing the activation of subsequent cell death effector molecules—key proteins integral to the initiation of inflammatory cell death responses, which encompass the NLRP3 inflammasome, CASP 8, and the RIPK1/RIPK3 complex. This cascade culminates in the occurrence of pyroptotic cell death [15].

#### Pyroptosis and PANoptosis

Pyroptosis signifies a destructive form of PCD elicited by the pore-forming actions of the gasdermin protein family. It is primarily activated through inflammasomes within the body, which in turn activate the caspase protein family for the cleavage and activation gasdermin proteins. Once activated, these gasdermin proteins relocate to the cell membrane, disturbing its structural integrity and culminating in the establishment of pores. This, in turn, prompts the outflow of cellular cytoplasm and the eventual demise of the cell [16]. The distinguishing morphological traits of pyroptosis include cellular swelling, rupture of the plasma membrane, and secretion of the inflammatory factors. The main signaling pathways of pyroptosis include the non-classical pathway (dependent on caspase-4, caspase-5, and caspase-11), the classical pathway (dependent on caspase-1 (CASP1)), as well as other pathways [17].

In the classical pyroptosis pathway, the precursor protein of CASP1 is capable of interacting with apoptosis-associated speck-like protein containing a caspase recruitment domain (ASC) and pattern recognition receptors (PRRs) to form an inflammasome. This inflammasome subsequently catalyzes the activation of CASP1, leading to the rupture of the plasma membrane and the discharge of pro-inflammatory cytokines (e.g., IL-1β and IL-18), ultimately resulting in cell death [18, 19]. PRRs associated with pyroptosis include NLRP1/3, Toll-like receptors (TLRs), AIM2-like receptors (ALR) [18], etc. In the non-classical pyroptosis pathway, cytoplasmic lipopolysaccharides (LPS) directly bind to caspase-4/5/11, thereby activating gasdermin proteins and causing plasma membrane dissolution, and ultimately triggering cell pyroptosis [20].

Relationship with apoptosis: pyroptosis stands as an inflammatory route to cell death, which is dependent on CASP1 activation. The NLRP3 released by PANoptosome triggers the production of pro-CASP1, which in turn orchestrates the formation of membrane pores by GSDMD and GSDMD proteins upon activation. This sequence of events leads to the release of inflammation and the execution of cell death [15].

#### Apoptosis and PANoptosis

Apoptosis is a PCD process mediated by caspase protein hydrolase under specific physiological or pathological conditions. Morphologically, apoptotic cells exhibit cellular crumpling, chromatin condensation and apoptotic vesicle formation [21, 22]. The main pathways that regulate cell apoptosis include endogenous, exogenous, and caspase non-dependent apoptotic signaling pathways [22].

There are specific mechanisms underlying cell apoptosis. The endogenous mitochondrial pathway is primarily modulated by the Bcl-2 family proteins. Under cellular stress, BH3-only proteins (which are part of the Bcl-2 family) act as important initiators of apoptosis by either increasing transcription or acting post-transcriptionally. This results in the activation of Bcl-2 effector proteins BAX and BAK, leading to the permeabilization of the outer mitochondrial membrane [23]. As a result, pro-apoptotic proteins are released from the mitochondria, and the released pro-apoptotic protein cytochrome c (Cty C) binds to the pro-apoptotic factor APAF1, leading to the formation of the apoptosome. This induces the activation of caspase-9 (CASP9), followed by downstream CASP3 and CASP7 activation. These caspases facilitate the proteolysis of numerous proteins, culminating in cell apoptosis [24]. The exogenous death receptor pathway primarily involves transmembrane death receptor family members such as tumor necrosis factor (TNF) receptor (TNFR), FS7-associated cell surface antigen (Fas) receptor, and TNF-related apoptosis-inducing ligand (TRAIL) receptors (DR4 and DR5) [11]. Activation occurs when these receptors bind to their respective ligands. The intracellular death domains present in these receptors lead to complex formation, initiating cell apoptosis and eventually activating CASP 8. This leads to the activation of downstream CASP3 and CASP7, promoting cell apoptosis [22].

The correlation between apoptosis and PANoptosis can be explained as follows: apoptosis is dependent on the release of CASP3 and CASP7 from apoptotic vesicles. In PANoptosis, CASP8, which is released by the PANoptosome, activates BH3 interacting-domain death agonist (BID), forming truncated BID. This process enhances mitochondrial outer membrane permeabilization in the mitochondria, resulting in the release of Cty C. Subsequently, Cty C contributes to the formation of apoptotic vesicles and the release of CASP3 and CASP7, further promoting apoptosis [15].

#### Necroptosis and PANoptosis

Necroptosis, also referred to as programmed necrosis, is an alternative PCD mechanism that occurs when the normal apoptotic pathway is inhibited. Unlike apoptosis, necroptosis occurs independently of caspases. Necroptosis is characterized by the morphological features of necrosis, such as the disruption of plasma membrane integrity, enlargement of cytoplasm and organelles, chromatin condensation, and production of cellular components such as damage-associated molecular patterns (DAMPs), pro-inflammatory cytokines, and chemokines. These events collectively trigger an inflammatory response within the organism [25]. Necroptosis can be mediated by members of the TNFR superfamily, including TNFR1, Fas, DR3 (i.e., TRAMP or APO-3), DR4, DR5, and DR6 [26]. Activation can also be induced by TLR3/4, interferon receptors, and ZBP1. Key players in the necroptosis-related signaling pathway encompass RIPK1, RIPK3, and MLKL [27]. Among them, RIPK3 phosphorylates MLKL and promotes its migration to the cell membrane by forming multimers as a key molecular mechanism of necroptosis.

The specific mechanism is as follows: conventional apoptosis hinges on caspase activation. When caspases undergo deficiency or their activity is suppressed, the standard apoptotic pathway is inhibited, potentially leading to the initiation of necroptosis as an alternative cell death pathway. In recent years, our comprehension of TNF-α-mediated necroptosis has advanced significantly. RIPK1 and RIPK3 are key molecules in the TNF-α-mediated necroptotic pathway. When TNF-α binds to TNFR on the cell membrane, RIPK1 is activated following cleavage. Activated RIPK1 facilitates downstream phosphorylation of RIPK3 and the expression of MLKL.

Necroptosis and PANoptosis: programmed necrosis, or necroptosis, is MLKL-dependent and independent of caspase mechanisms. The release of upstream RIPK1/RIPK3 complexes from the PANoptosome triggers MLKL phosphorylation and the assembly of MLKL proteins, ultimately culminating in cellular necrosis [15].

In summary, the formation of the PANoptosome seems to be highly contingent on specific triggers and may be modulated by various pathogens and DAMPs encountered during disease. Thus, identifying novel triggers and sensors is pivotal in advancing our comprehension of this distinctive form of cell death.

### Interconversion of different PCD modalities

#### Apoptosis and necroptosis

The pivotal molecule responsible for maintaining a balance between apoptosis and necroptosis is CASP8, along with its downstream counterpart RIPK1. Mice lacking CASP8 experience embryonic mortality [28], while knockout of RIPK3 or MLKL, the key molecule of necroptosis, mitigates their fatal effects [29]. This implies that CASP8 plays a significant role in curbing necroptosis. Mice with RIPK1 knockout postnatal mortality, which might be attributed to extensive necroptosis in epithelial cells and apoptosis in intestinal cells [30]. The concurrent depletion of both CASP8 and RIPK3 suppresses both apoptosis and necroptosis, ultimately rescuing RIPK1-deficient mice from demise [31]. The induction of RIPK1 in different PCD processes is correlated with its expression, in which lower expression favors apoptosis, while upregulated expression inclines toward provoking necroptosis, often leading to necrotic apoptosis [32].

#### Pyroptosis and necroptosis

RIPK3-MLKL-mediated necrotic apoptosis engages with the activation of inflammatory vesicles. Knockdown of CASP8 or inhibition of its function promotes the initiation of NLRP3, a process facilitated by either RIPK3 alone or the involvement of MLKL/phosphoglycerate mutase 5 [33, 34]. This suggests that necroptosis promotes cellular scorching. Previous research has found that MLKL induces the activation of inflammatory vesicles by promoting ASC oligomerization, which is dependent on the MLKL death effect quadruple helix bundle, MLKL oligomerization and its interaction with the cell membrane, as well as a reduction in intracellular potassium ion content [35]. Elevating the concentration of extracellular potassium ions effectively inhibits MLKL-mediated activation of NLRP3 [35].

#### Apoptosis and pyroptosis

In 2008, researchers initially identified CASP1 in macrophages as being responsible for cleaving the conventional aspartate activation site of CASP7. This phenomenon was observed when microbial stimulation triggered the activation of CASP7, and silencing CASP1 effectively suppressed this activation [36]. When GSDMD is absent, CASP1 proceeds to cleave CASP3, thereby triggering the apoptotic signaling pathway [37]. As a result, in cells containing minimal or no GSDMD content, CASP1 takes the lead in promoting apoptosis as a means of inducing cellular demise. At the same time, apoptosis is involved in regulating the activation of inflammatory vesicles. Directly interacting with the abasicin protein, CASP3 becomes activated. Once activated, it cleaves GSDMD, initiating pyroptosis, a process that can result in tissue damage [38]. In contrast, when the cytotoxic N-terminal segment of GSDMD is cleaved, it generates an inactive fragment that inhibits pore formation [39].

## PANoptosis in breast cancer

Under normal physiological conditions, a process known as RCD takes place, which is also referred to as PCD. There are various recognized forms of RCD, and each mode of RCD is initiated and propagated through interconnected molecular mechanisms. Moreover, each specific type of RCD can exhibit a range of structural features, spanning from complete cell necrosis to full-fledged apoptosis. These forms of RCD also possess immune-regulating attributes that encompass anti-inflammatory and tolerogenic responses, as well as pro-inflammatory and immunogenic responses. During the course of RCD, different pathways that induce cell demise can influence cancer progression and treatment effectiveness. In the initial stages of the disease, cancer cells might demonstrate resistance to anti-cancer therapies owing to genetic mutations that disrupt the pathways associated with RCD. Avoidance of RCD is considered a key characteristic of cancer. Recent research has highlighted multiple factors linked to RCD that play a role in the development and advancement of triple-negative breast cancer (TNBC) [9]. For instance, He et al. [40], using an in-depth analysis of breast cancer profiles through the utilization of TCGA and GEO databases, found that high expression of PANoptosis was conducive to reducing the incidence of breast cancer.

### Pyroptosis in breast cancer

Pyroptosis assumes a dual role in the progression of diverse types of tumors. Inflammatory vesicles, with the NLRP3 inflammatory vesicle being the most extensively studied among them, can affect both the occurrence of tumor cells and the composition of the tumor microenvironment. The induction of cell pyroptosis and activation of the inflammasome can facilitate the demise of tumor cells, suppressing their proliferation and impeding metastasis. Conversely, inflammasome activation contributes to the formation of a tumor microenvironment conducive to the growth, propagation, and metastasis of tumor cells. Abnormal inflammasome activation is correlated with tumor growth and the acceleration of metastasi [41–43]. Research has unveiled that inflammatory bodies can galvanize the body's immune response and act as a deterrent against cancer occurrence [44]. Moreover, the pro-tumor effect of miR-233 might be intertwined with its capability to inhibit NLRP3 inflammasome activation. The genetic locus of miR-233 plays a role in promoting cancer cell growth and angiogenesis [45], and the elevated expression of NLRP3 after miR-233 depletion in breast cancer cells suggests other possibilities for activating NLRP3 against tumors [46]. Furthermore, knocking out the NLRP3 inflammasome increases the apoptotic rate of MCF-7 breast cancer cells [47].

Instigating pyroptosis can lead to atypical inflammasome activation, especially involving the NLRP3 inflammasome, which exerts a promotive effect on tumor inception and metastasis. GSDMD is a pivotal protein responsible for releasing inflammatory factors during pyroptosis [48]. Research has indicated that GSDMD is highly expressed in breast cancer patients with a HER2-positive status, correlating with diminished response to breast cancer chemotherapy and poorer prognosis. High GSDMD expression is correlated with low survival rates and high metastasis rates, indicating low responsiveness to HER2-targeted therapy [49]. Therefore, targeting the GSDMD protein could hold promise as an effective therapeutic strategy. In tandem, inflammasome activation and the involvement of the GSDMD protein reshape the tumor microenvironment by influencing the secretion and release of the ultimate products of pyroptosis, namely IL-1β and IL-18. These molecules recruit distinct subsets of immune cells to suppress tumor immunity and promote tumor development.

Prior investigation has found that IL-1β can positively regulate the self-renewal of cancer stem cells, thereby expediting tumor growth and invasion [50]. Elevated levels of IL-1β have been detected in a variety of solid tumors, including breast cancer, lung cancer, colorectal cancer, and melanoma [51]. Pizato et al. [52] discovered that when they treated MDA-MB-231 TNBC cells and 4T1 TNBC cells with docosahexaenoic acid (DHA), these cells were capable of releasing more cellular markers associated with cellular pyroptosis. Additionally, DHA treatment caused the translocation of HMGB1 from the cell nucleus to the cytoplasm by activating caspase-1 and GSDMD. This activation of caspase-1 and GSDMD not only relocated HMGB1 but also increased the activity of caspase-1 and GSDMD, leading to the secretion of IL-1β. Ultimately, this process induced cell death, confirming the anticancer effect of DHA and shedding light on its underlying mechanism. Moreover, Wang et al. [53] demonstrated that eliminating less than 15% of tumor cells through necroptosis was adequate to eradicate the entire 4T1 breast tumor graft. Importantly, the extent of tumor regression correlated with an enhanced anti-tumor immune response, which was absent in immune-deficient mice or under T cell-depleted conditions. This emphasizes the potential of precisely modulating inflammasome activation and necroptosis to significantly enhance the effectiveness of immune-based therapies.

Consequently, there exists a need for further exploration into clinical pathways of targeted cell necroptosis, inhibitors of inflammatory bodies that regulate tumor growth, and monoclonal antibodies. In addition to directly inducing tumor cell necroptosis, a growing body of evidence suggests that cell necroptosis holds promise as an immunotherapeutic avenue to enhance systemic cancer treatment. The immunogenicity of cancer cells represents a fresh trajectory in tumor therapy.

### Apoptosis in breast cancer

The halt of apoptosis stands as a prominent hallmark of malignant tumors, including breast cancer. The aberrant molecular mechanism of the apoptosis signaling pathway in breast cancer cells, which induces their re-entry into the apoptotic cycle, may be a crucial approach for treating breast cancer. The tumor suppressor gene p53 maintains genomic stability in normal human tissues under various stress conditions and is markedly related to the occurrence, development, and treatment of cancer. Compound G613 can promote apoptosis in MCF-7 cells and inhibit tumor growth in transplanted mice by inhibiting the formation of the p53-MDM2 complex and increasing p53 expression [54]. Bcl-2, Mcl-1, and Bcl-xL, members of the Bcl-2 family, serve as anti-apoptotic agents. Conversely, Bax, Bak, and Bim, which belong to the Bcl-2 family, exert a pro-apoptotic role. The balance between these two groups may influence the occurrence, development, and treatment of cancer. Clinical observations have revealed that approximately 75% of breast cancer tissues exhibit elevated Bcl-2 expression [55]. Hence, Bcl-2 expression can serve as an indicator for determining the sensitivity of breast cancer to chemotherapy [56]. The Bcl-2 protein family plays a crucial regulatory role in regulating apoptosis during the treatment of breast cancer. A specific small molecule inhibitor of Bcl-2 known as ABT737 can induce apoptosis and hinder growth in radiation-resistant cells, such as MB231R, by inhibiting Bcl-xL or Mcl-1 [57, 58]. Aspirin can induce Bcl-2 phosphorylation, resulting in the formation of a complex with FKBP38 protein that enters the cell nucleus and induces apoptosis in MCF-7 cells [59]. BH3 mimetics, which specifically target Bcl-2, have strong efficacy and minimal side effects. These compounds can easily permeate cell membranes, efficiently counteracting the activity of Bcl-2 cancer-associated proteins. Clinical trials focusing on BH3 mimetics (e.g., ABT737/199) in combination with standard breast cancer therapeutic drugs (e.g., tamoxifen) will undoubtedly promote the utilization and progression of the Bcl-2 apoptotic pathway in breast cancer treatment.

CASP8, a key player in the enzymatic cascade leading to apoptosis, plays a pivotal role in this cellular process. Ao et al. [60] investigated the inhibitory effect of active CASP8 (Gag-CASP8-VLPs) delivered via HIV Gag virus-like particles (VLPs) conjugated with CASP8 (Gag-CASP8-VLPs), on breast cancer growth. Their findings revealed that Gag-CASP8-VLPs effectively transported CASP8 to breast cancer cells, inducing apoptosis and impeding tumor growth. Lan et al. [61] explored the targeted anti-breast cancer effects and the underlying mechanisms by constructing folic acid albumin nanoparticles loaded with baicalin (FA-BSANPs/BA). Their research unveiled that FA-BSANPs/BA facilitated apoptosis by upregulating caspase-8 expression and increasing the levels of ROS while concurrently reducing the level of Bid.

To sum up, the study of apoptosis-related signaling pathways and molecular mechanisms, the identification of apoptosis mediators, and the exploration of modulators and targeted drugs associated with these mechanisms hold promise for the advancement of new approaches and medications in the treatment and prevention of breast cancer.

### Necroptosis in breast cancer

Numerous studies have provided compelling evidence regarding the significant role of necroptosis in both the development and treatment of breast cancer. Under normal circumstances, necroptosis is a process of cellular self-destruction, a mechanism by which cells respond to various signals of injury. However, the regulatory mechanism of breast cancer cells is disrupted, enabling them to proliferate endlessly and circumvent the typical cellular death pathway. It has been demonstrated that targeting necrotic proteins is essential for breast cancer development and that cellular resistance to necrotic regulation is often mediated by oncogenes, suggesting that evading necrotic regulation might be a potential hallmark of tumors, akin to evading cellular regulation [62]. Choi et al. [63] found that aurora kinase (AURK) binds to IκBα in the cell and identified two new serine phosphorylation sites, namely, S63 and S262. These phosphorylation sites, along with AURK and IκBα, were found to be associated with the necroptotic apoptotic pathway in breast cancer cells. Consequently, necrotic apoptosis in tumor cells leads to tumor necrosis and promotes tumor metastasis. Karsch-Bluman et al. [64], in their investigation of MDA-MB-231 cells, found that the presence of necrotic cells promoted angiogenesis, vascular endothelial cell proliferation, enhanced cell invasive migration, and cell–cell interactions.

Tumor necrosis is commonly observed in the core region of solid tumors once they reach a certain size. This necrosis is often accompanied by a decrease in the expression of key necrosis-related molecules, such as RIPK3, in breast cancer cell lines (e.g., MDA-MB-231 and MCF-7). Clinical studies conducted by Koo et al. [65] demonstrated that RIPK3 expression was reduced in the tumors of 85% of breast cancer patients when compared to that of the normal group. This suggests that necrotic apoptosis plays an inhibitory role in the growth and development of breast cancer. Shen et al. [66] explored the role of RIP1/RIP3/MLKL signaling in the proliferation and metastasis of breast cancer cells both in vivo and in vitro. Their findings indicated that modulating necroptosis might represent a novel therapeutic approach for breast cancer. In addition, RIPK3-mediated phosphorylation of MLKL can lead to MLKL oligomerization and its subsequent translocation to the plasma membrane. Jiao et al. [67] found that MLKL expression significantly increased in tumor cells during tumor bullous growth, with phosphorylated MLKL specifically detected in cells surrounding the necrotic region. Long-stranded non-coding RNAs (IncRNAs) are key regulators in breast cancer. Zhang et al. [68] predicted that lncRNAs associated with necroptosis might determine the prognosis of breast cancer patients. They constructed a prognostic model based on the expression profiles of these lncRNAs, which could potentially aid in evaluating patient prognosis, response to immunotherapy, and serve as promising therapeutic targets.

Necrotic apoptosis genes have been implicated in the pathogenesis and progression of breast cancer, playing a dual role in malignant cancers, including breast cancer. They are believed to influence the prognosis of breast cancer patients and their response to drug therapy. Thus, further exploration of the details of necrotic apoptosis pathways as a novel mode of cell death is ongoing. Understanding the role of these genes in breast cancer may help identify more precise and effective therapeutic targets.

## Conclusion and future prospect

Breast cancer currently stands as the most prevalent malignant tumor affecting women globally, and its frequency and fatality rates surge yearly in tandem with population growth and aging. Despite the ongoing advancement in medical knowledge, the precise triggers and mechanisms responsible for breast cancer remain unidentified, and the overall outcomes of treatment fall short of the desired mark. Hence, it becomes imperative to uncover novel therapeutic targets in order to enhance patient prognosis and survival rates. PCD, including apoptosis, necroptosis, and pyroptosis, emerges as closely intertwined with both the occurrence and prognosis of breast cancer. Within this landscape, a distinctive form of inflammatory cell death called PANoptosis is regulated by the death-inducing PANoptosome complex. It exhibits cardinal attributes of apoptosis, necroptosis, and pyroptosis, without being exclusively confined to any single mode of demise. Moreover, diverse forms of PCD associated with PANoptosis can undergo interconversion. The interplay between PANoptosis and tumors can be categorized into three groups: cell pyroptosis and breast cancer, cell necroptosis and breast cancer, which play a dominant role in promoting cell death (synergistic or cooperative action); cell apoptosis and breast cancer by inducing apoptosis to promote cell death (promoting action); and cell necroptosis antagonizing or inhibiting apoptosis-induced cell death in breast cancer (antagonistic or inhibitory action).

Although has been substantial research into the roles of apoptosis, necroptosis, and pyroptosis in the context of breast cancer, it is worth noting that the exploration of PANoptosis in breast cancer remains relatively limited in terms of available literature. Currently, our understanding of the molecular mechanisms governing PANoptosis and the composition of the PANoptosome is largely confined to a handful of pivotal regulatory targets, such as the upstream molecules ZBP1 and RIPK1, AIM2, caspase family members (including CASP3, CASP6, and CASP8), and interferon regulatory factors 1 as downstream components. These molecules have the capacity to trigger the assembly of the PANoptosome in response to specific stimuli. However, it is likely that there are additional, as-yet-uncharacterized molecules involved in this process. The identification of these markers promises to herald transformative advancements in refining future strategies for cancer treatment. Hence, forthcoming research endeavors will be focused on identifying more PANoptosis-inducing sensors and comprehensively delineating the constituents of the PANoptosome in correlation with various aseptic and pathogenic stimuli.

This comprehensive review systematically elucidates the definition and mechanisms of PANoptosis, the intricacies of interchanging various forms of PCD associated with PANoptosis, and the interconnections between breast cancer and PCD. It serves as a complement and guide to the research on “PANoptosis in breast cancer.” In summary, despite significant strides in breast cancer treatment, the mortality rate remains alarmingly high. The origins of breast cancer are intricate, as different patients exhibit distinct pathogenic mechanisms, and the factors contributing to apoptosis resistance remain enigmatic. In light of these challenges, this review places its focus on establishing the relationship between PANoptosis and cancer, the role it plays in mediating tumor cell death, and its potential to surmount obstacles hindering effective cancer treatment. The interaction between PANoptosis and breast cancer poses a newly emerging research question for investigators. The proactive identification of personalized biomarkers and responsive treatment targets, coupled with the discovery of optimal treatment strategies, becomes a pivotal avenue for successfully addressing breast cancer. Although the realm of precise molecular targeted therapy for breast cancer is still at an early stage, the convergence of genome sequencing and the emergence of the big data era call for intensified exploration of potential molecular targets within key apoptosis signaling pathways. This exploration aims to pave the way for combination therapies targeting multiple facets.

In conclusion, this review makes a concerted effort to underscore the interplay between PANoptosis and diverse cell death pathways in the context of breast cancer, as well as the pivotal role of these interactions in the progression of cancer. It is hoped that this review will provide a solid groundwork for researchers in this field to explore the potential mechanisms of PANoptosis in breast cancer in the near future.

## Data Availability

Not applicable.

## Abbreviations

AML: Acute myeloid leukemia
AIM2: Absent in melanoma 2
ALR: AIM2-like receptors
BC: Breast cancer
BID: BH3 interacting-domain death agonist
CASP1/3/7/8: Caspase-1/3/78
Cty C: Cytochrome C
DAMPs: Damage-associated molecular patterns
DHA: Docosahexaenoic acid
Fas: FS7-associated cell surface antigen
HER2: Human epidermal growth factor receptor 2
GSDMD: Gasdermin D
IAV: Influenza A virus
LPS: Lipopolysaccharides
MLKL: Mixed lineage kinase domain-like
NLRP3: NLR Family Pyrin Domain Containing 3
PCD: Programmed cell death
PRRs: Pattern recognition receptors
RCD: Regulatory cell death
RIPK1/3: Receptor Interacting Serine/Threonine Kinase 1/3
RHIM: RIP homotypic interaction motif
TAK1: TGF-β-activated kinase 1
Tbid: Truncated BH3 interacting-domain death agonist
TLRs: Toll-like receptors
TRAIL: TNF-related apoptosis-inducing ligand
TCB: Trichlorobenzene sulfonamide
TNBC: Triple-negative breast cancer
TNF: Tumor necrosis factor
TNFR: Tumor necrosis factor receptors
ZBP1: Z-DNA Binding Protein 1
